# Chromosome-Level Alpaca Reference Genome *VicPac3.1* Improves Genomic Insight Into the Biology of New World Camelids

**DOI:** 10.3389/fgene.2019.00586

**Published:** 2019-06-21

**Authors:** Mark F. Richardson, Kylie Munyard, Larry J. Croft, Theodore R. Allnutt, Felicity Jackling, Fahad Alshanbari, Matthew Jevit, Gus A. Wright, Rhys Cransberg, Ahmed Tibary, Polina Perelman, Belinda Appleton, Terje Raudsepp

**Affiliations:** ^1^Genomics Centre, Deakin University, Geelong, VIC, Australia; ^2^Centre for Integrative Ecology, Deakin University, Geelong, VIC, Australia; ^3^School of Pharmacy and Biomedical Sciences, Curtin Health Innovation Research Institute, Curtin University, Perth, WA, Australia; ^4^Bioinformatics Core Research Group, Deakin University, Geelong, VIC, Australia; ^5^Department of Genetics, The University of Melbourne, Melbourne, VIC, Australia; ^6^Department of Veterinary Pathobiology, Texas A&M University, College Station, TX, United States; ^7^Center for Reproductive Biology, Washington State University, Pullman, WA, United States; ^8^Institute of Molecular and Cellular Biology, Siberian Branch of Russian Academy of Sciences, Novosibirsk, Russia

**Keywords:** alpaca, reference genome, *VicPac3.1*, chromosome-level, Dovetail Chicago, MHC, *Minute Chromosome Syndrome*, high-altitude adaptations

## Abstract

The development of high-quality chromosomally assigned reference genomes constitutes a key feature for understanding genome architecture of a species and is critical for the discovery of the genetic blueprints of traits of biological significance. South American camelids serve people in extreme environments and are important fiber and companion animals worldwide. Despite this, the alpaca reference genome lags far behind those available for other domestic species. Here we produced a chromosome-level improved reference assembly for the alpaca genome using the DNA of the same female Huacaya alpaca as in previous assemblies. We generated 190X Illumina short-read, 8X Pacific Biosciences long-read and 60X Dovetail Chicago^®^ chromatin interaction scaffolding data for the assembly, used testis and skin RNAseq data for annotation, and cytogenetic map data for chromosomal assignments. The new assembly *VicPac3.1* contains 90% of the alpaca genome in just 103 scaffolds and 76% of all scaffolds are mapped to the 36 pairs of the alpaca autosomes and the X chromosome. Preliminary annotation of the assembly predicted 22,462 coding genes and 29,337 isoforms. Comparative analysis of selected regions of the alpaca genome, such as the major histocompatibility complex (MHC), the region involved in the *Minute Chromosome Syndrome* (MCS) and candidate genes for high-altitude adaptations, reveal unique features of the alpaca genome. The alpaca reference genome *VicPac3.1* presents a significant improvement in completeness, contiguity and accuracy over *VicPac2* and is an important tool for the advancement of genomics research in all New World camelids.

## Introduction

Alpacas and llamas were domesticated in the high Andes around 9,000 years ago and have been associated with humans for as long as cattle, horses and dogs (Wheeler, 1995; Bruford et al., 2003). It is thought that the ancient Incan civilization owed success largely to llama dung, which provided fertilizer and enabled corn to be cultivated at very high altitudes. Today, alpacas continue to serve the rural families of the Altiplano as an important source of fiber and meat (Cruz et al., 2017). In addition, alpacas are also gaining popularity worldwide, mainly for their high quality fiber, and as a docile companion species. In addition, alpacas and camelids in general, are species of broader interest for several fields in biology and biomedical sciences. For example, the family Camelidae forms the most basal clade in the phylogeny of the eutherian order Cetartiodactyla (Murphy et al., 2005; Zhou et al., 2011) and is, thus, a key-group in the mammalian evolutionary tree, and is being used to aid in the annotation of the human genome (Genome 10K Community of Scientists, 2009). Further, genetic relationships between South American camelids, the domesticated alpaca (*Vicugna pacos*) and llama (*Lama glama*), and the wild guanaco (*Lama guanicoe*) and vicuña (*Vicugna vicugna*), are intriguing and still not completely resolved (Bruford et al., 2003; Barreta et al., 2013; Marin et al., 2018). All camelids are uniquely adapted to extreme environments – the New World species to high altitude and the Old World camels to arid desert environments (Wu et al., 2014), due to these adaptations their genomes may reveal important signatures of natural or human selection. Camelids are also of biomedical interest because of the presence of small and functionally efficient heavy chain-only antibodies, which are not found in other mammalian groups (Flajnik et al., 2011; Griffin et al., 2014; Cohen, 2018).

Despite being a species of broad interest, the analysis of camelid genomes, including that of the alpaca, had a late start and lags behind other domesticated species. Camelid karyotypes were described in the 1980s (Bianchi et al., 1986), showing that all extant species have a conserved diploid number (2n = 74) and very similar chromosome morphology. Yet, the first cytogenetic and comparative chromosome maps for these species emerged only recently (Balmus et al., 2007; Avila et al., 2014a,b, 2015), almost concurrently with genome sequencing projects. At present, there are two annotated sequence assemblies for the alpaca that are available at all main Genome Browsers such as NCBI^1^, UCSC^2^ and Ensembl^3^: *VicPac1* (version 1.0) and *VicPac2* (version 2.0.1). Both used DNA from the same female Huacaya individual. The first assembly was generated at the Broad Institute by Sanger sequencing and has 2.51X genome coverage, the second was assembled at Washington University by combining the former Sanger reads with newly generated 454 GS FLX data. This resulted in an assembly with 22X genome coverage and annotation for 24,553 genes and 33,208 proteins. *VicPac1* and *VicPac2* form the alpaca reference genome and are currently the main tools for alpaca genomics. There is also a third assembly, *Vipacos_V1.0*, which was generated for the comparison of genomic signatures of selection and adaptations between the dromedary, Bactrian camel and alpaca (Wu et al., 2014). *Vipacos_V1.0* was assembled from short-read Illumina data and reached 72.5X genome coverage, but is not integrated with *VicPac1* or *VicPac2*. Despite this progress, all three alpaca assemblies are relatively short – 2 billion DNA base-pairs (2 Gb) instead of the anticipated 2.5–3 Gb; all are fragmented into a large number of contigs and scaffolds, and none have scaffolds assigned to chromosomes. The overall utility of these datasets as an alpaca reference genome to serve the interests of researchers, breeders and the health and welfare of the animals, is therefore limited and needs improvement.

The aim of this study was to re-sequence, re-assemble *de novo* and re-annotate the alpaca genome using the same female Huacaya DNA donor as in *VicPac1* and *VicPac2*. We used next generation long- and short-read sequencing platforms to generate the data and initial assembly; Dovetail Chicago^®^ scaffolding and HiRise^TM^ for advanced assembly; RNAseq and bioinformatics pipelines for annotation, and cytogenetic comparative map data to anchor sequence scaffolds to chromosomes.

## Results and Discussion

### Genome and Assembly Features

The genome of a female Huacaya alpaca was sequenced generating ∼190X genome coverage of paired-end (PE) and mate-pair (MP) short-read Illumina data (2.72 billion PE reads, 272 Gb; 1.52 billion MP reads, 152 Gb), ∼8X genome coverage of Pacific Biosciences (PacBio) long-read data (2.4 million subreads; 18.0 Gb), and ∼60X genome coverage Dovetail Chicago^®^ chromatin interaction scaffolding data (459 million PE reads; 137.7 Gb). A multi-stage assembly improvement strategy was applied through four separate assembly iterations. Firstly, we produced a hybrid *de novo* assembly using the PE and MP short-read data together with the Sanger and 454 data from the *VicPac1* and *VicPac2* assemblies, respectively. This assembly (*Qmas1*) had more contigs and scaffolds than *VicPac2*, lower scaffold N50, but higher contig N50 (Table 1). Next, we integrated *Qmas1* and *VicPac2* to produce a meta-assembly (*Qmas1/VicPac2*) that resulted in contiguity improvements, namely a reduction in the number of contigs and scaffolds and the simultaneous increase in contig and scaffold N50s (Table 1). The next iteration of the assembly incorporated the ∼ 8X PacBio long-read data and resulted in modest improvements to the assembly (designated *VicPac3*) compared to previous iterations (Table 1). The final assembly iteration involved scaffolding the *VicPac3* assembly with the MP short read data and Dovetail Chicago^®^ data. This final assembly also resulted in significant improvements in the assembly metrics (see Table 1), including a significant increase of scaffold N50 from 9.86 Mb in *VicPac3* to 24 Mb in *VicPac3.1*. Compared to all previous assemblies, *VicPac3.1* has the best assembly metrics and most importantly, 90% of the assembly sequence length (L90) is contained in just 103 scaffolds (0.1% of all scaffolds; Table 1). The remaining 10% of the assembly sequence length is made up of smaller, fragmented scaffolds. Addition of higher coverage long-read data, for example 20X, compared to the 8X we used, may be needed to generate further improvements to the assembly, through filling gaps and joining scaffolds. The most critical improvements in the contiguity and accuracy of the assembly occurred during the meta-assembly of *Qmas1* and *VicPac2*, and subsequent HiRise^TM^ scaffolding of *VicPac3*. The latter corrected 240 inaccurate assemblies, joined 1813 scaffolds, and essentially improved the size of scaffold N50 and reduced L50 and the total number of scaffolds (Table 1).

**Table 1:** Comparative summary statistics of alpaca genome assemblies.

	*VicPac3.1*	*VicPac3.0*	*Qmas1/VicPac2*	*Qmas1*	*VicPac2.0*	*VicPac1.0*	*Vipacos_V1.0*
Breed	Huacaya	Huacaya	Huacaya	Huacaya	Huacaya	Huacaya	Huacaya
Sex	Female	Female	Female	Female	Female	Female	Female
Individual	*Carlotta*	*Carlotta*	*Carlotta*	*Carlotta*	*Carlotta*	*Carlotta*	n/a
Assembly size (Gb)	2.12	2.12	2.12	2.66	2.17	2.96	2.01
Contig N50 (kb)	35.72	35.75	35.75	306.09	29.07	3.91	66.3
Number of contigs	204,817	204,577	205,666	719,860	412,904	721,292	75,733
Scaffold N50 (Mb)	24.02	9.86	9.06	5.83	7.26	0.23	5.1
Scaffold L50	25	64	69	126	86	2,595	–
Number of scaffolds	77,390	78,963	82,481	678,087	276,726	298,413	4,322
Longest scaffold (Mb)	121.37	38.36	38.36	25.07	38.45	5.51	–
GC %	41.4	41.4	41.4	41.6	41.4	39.7	41.5
N’s %	4.17	4.09	3.98	2.44	4.31	35.09	–
Repeat %	33.48	–	–	–	34.74		32.1
Reference	This study	This study	This study	This study	NCBI^a^,	NCBI,	Wu et al., 2014
					UCSC^b^,	UCSC,
					Ensembl^c^	Ensembl


*Source: ^*a*^https://www.ncbi.nlm.nih.gov/, ^*b*^https://genome.ucsc.edu/, ^*c*^http://www.ensembl.org/index.html.*

The GC-content of the alpaca genome was ∼41% and remained the same across all our assembly iterations, and is similar to that reported in prior alpaca assemblies (Table 1). The 2.12 Gb size of the re-assembled genome *VicPac3.1* is similar to previous assemblies of the same individual (Table 1) but smaller than the 2.63 Gb estimation by k-mer analysis (Wu et al., 2014). Genome size estimation using a range of k-mer frequencies obtained from our short-read data produced size estimates ranging from 2.05 to 2.29 Gb (Supplementary Figure 1 and Supplementary Table 1), which are very similar to the obtained genome sizes for all assemblies in Table 1 for the same animal, but smaller than the prior k-mer estimation (Wu et al., 2014). On the other hand, measurement of the genome size by flow cytometry using alpaca fibroblasts suggested size of 2.88 Gb with a range of 2.73–3.01 Gb (95% confidence interval; Supplementary Figure 2), thus larger than the bioinformatic estimates by us or others. However, it must be noted that the available computational and empirical methods for estimating genome size are subject to very large errors. Furthermore, genome size will vary between individuals. These factors combined may account for the differences between the estimates, and the exact size of the alpaca genome is yet to be determined by additional studies.

The Benchmarking Universal Single-Copy Orthologs (BUSCO)^4^ mammalian gene set with 4,104 conserved mammalian orthologs (hereafter BUSCOs) was used to assess genome completeness in terms of recovery of these BUSCOs, to evaluate assembly iterations and compare them to previous alpaca assembly versions. While BUSCO analysis is more appropriate for direct comparison of different genome assemblies within a species, it can provide useful benchmarks when compared to assemblies of other species. Therefore, we also produced BUSCO assessment data for cow, sheep, dromedary and Bactrian camel. Serial improvements in BUSCO scores were observed throughout the iterative assembly process (Table 2), with the final assembly, *VicPac3.1*, having the highest BUSCO completeness at 96.1% with 3,944 genes and the lowest number of missing BUSCOs (77 genes; 1.9%). Compared to other available camelid genomes, the final assembly demonstrated comparable, but slightly superior scores across all metrics, suggesting that this assembly is one of the most complete available for camelids, and has completeness scores comparable with the cattle and sheep genomes. The datasets are available in BioProject ID PRJNA544883.

**Table 2:** BUSCO analysis of genome completeness.

	Complete and single copy (%)	Complete and duplicated (%)	Fragmented (%)	Missing (%)
**Alpaca genomes**				
*VicPac3.1*	96.1	0.7	2.0	1.9
*VicPac3.0*	94.7	0.7	2.4	2.2
*Qmas/VicPac2*	95.0	0.7	2.1	2.2
*Qmas1*	94.2	0.8	2.1	2.9
*VicPac2.0.2*	93.9	0.8	2.6	2.7
**Other camelid genomes**				
*Camelus dromedarius*	95.0	0.5	2.5	2.0
*C. bactrianus ferus*	94.5	1.2	2.6	1.7
*C. bactrianus*	95.2	0.5	2.3	2.0
**Select mammalian genomes**				
*Bos taurus*	92.4	1.2	3.0	3.4
*Ovis aries*	92.1	1.1	3.4	3.4


### Chromosomal Assignment

Sequences from the available alpaca cytogenetic map (Avila et al., 2014a,b, 2015) and comparative data with human, cattle and pig genomes (Balmus et al., 2007) were used to anchor the alpaca genome sequence assembly to physical chromosomes. In *VicPac3.1*, 75.9% of sequence scaffolds (in bp; ∼1.6 Gb) are mapped to the 36 pairs of alpaca autosomes and the X chromosome (Table 3 and Supplementary Table 2) providing the first chromosome-level assembly for the alpaca, or any camelid genome. Notably, this is a 31.9% increase in the amount of anchored sequence compared to our anchoring of *VicPac2* (44% of sequence scaffolds in bp; 0.96 Gb). Among the most notable improvements are assemblies of 14 alpaca chromosomes, *viz.*, chrs2, 5, 7, 8, 10, 17, 19, 22, 24, 27, 28, 31, 33, and 34 that uniquely correspond to a single large scaffold (Table 3); *VicPac2* only contains 2 chromosomes made up of single scaffolds (chrs19 and 31; Table 3 and Supplementary Table 3). Additionally, the total number of chromosomally anchored scaffolds was reduced from 129 in *VicPac2* to 88 in *VicPac3.1*, while simultaneously increasing the percentage of the genome anchored, further highlighting the significant improvements in contiguity of *VicPac3.1*. Currently, the most contiguous and largest is the 121 Mb scaffold of chr2, which likely represents the entire chromosome. In contrast, chr11 and chr16 remain rather fragmented and correspond to six different scaffolds each. It is notable that three scaffolds, with a total size of 5 Mb, correspond to the smallest autosome, chr36, because no sequences were assigned to this chromosome by Zoo-FISH (Balmus et al., 2007). Despite this progress, assemblies of several large chromosomes remain fragmented and incomplete. For example, eight unique scaffolds correspond to chrX, but cover collectively less than 40 Mb of the anticipated 150 Mb, which is the size of an average mammalian X chromosome^5^^,^^6^^,^^7^.

**Table 3:** Chromosomal assignment of *VicPac3.1* showing per each chromosome assembly size, number of unique assigned scaffolds, number of annotated genes, gene density, and human homology.

	*VicPac3.1*					*VicPac2.0.2*	
							
Alpaca chr.	Assembly size, bp	No. of unique mapped scaffolds	No. of genes	Genes per Mb	Human homology	Assembly size, bp	No. of unique mapped scaffolds
1	101,041,233	5	1625	16.1	3q, 21q	41,153,578	5
2	121,370,620	1	1650	13.6	4	36,264,523	4
3	83,363,794	3	1269	15.2	5	66,866,246	7
4	65,636,945	3	1166	17.8	9	36,674,619	6
5	96,274,254	1	1428	14.9	2q	67,623,744	5
6	74,791,714	2	1448	19.6	14q, 15q	34,188,095	5
7	31,168,711	1	641	2.1	7	9,531,993	3
8	70,270,077	1	1028	14.7	6q	62,544,616	5
9	74,791,714	3	1081	14.4	1p, 16q, 19q	26,984,682	4
10	39,582,034	1	794	19.9	11	0	0
11	77,176,758	6	1958	25.4	1q, 10q	36,454,531	6
12	48,986,614	2	910	18.6	12q	30,043,790	2
13	61,008,235	3	1491	2.4	1p	55,336,466	6
14	67,111,318	2	901	13.4	13q	19,497,535	4
15	32,418,436	2	643	2.0	2p	23.521.627	3
16	39,074,364	6	1220	3.1	10p, 17q	36,989,750	8
17	46,944,759	1	887	18.9	3p	40,598,934	2
18	29,910,177	2	930	31.0	7, 16p	24,952,824	2
19	24,022,313	1	693	28.9	20q	12,494,946	1
20	38,741,345	2	1110	28.5	6p	15,672,241	2
21	29,520,914	3	895	30.9	1q, 16q^∗^	15,741,292	4
22	25,522,599	1	891	34.3	5q, 19p	26,415,928	2
23	29,440,657	2	520	17.9	1q, 13q	32,337,675	3
24	18,346,407	1	318	17.7	18	15,189,407	2
25	60,195,357	3	1467	24.5	8q	26,317,781	5
26	27,987,978	2	537	19.2	4q, 8p	32,483,939	2
27	22,699,463	1	11	0.5	15q	8,774,263	2
28	16,162,605	1	412	25.8	2p	13,182,695	2
29	16,162,605	2	461	28.8	8q	24,598,565	2
30	13,130,742	3	238	18.3	18q	11,278,592	3
31	13,602,737	1	323	23.1	4, 8p	12,583,175	1
32	22,732,685	3	677	29.4	12q, 22q	8,370,595	3
33	16,261,182	1	451	28.2	11q	12,417,884	2
34	22,097,801	1	526	23.9	12p	16,301,232	2
35	18,484,027	4	397	22.1	10p	10,673,185	4
36	5,377,765	3	64	12.8	7p	3,455,596	4
X	36,971,808	8	687	17.2	X	16,549,574	6


*^∗^Denotes novel conserved synteny, not detected by Balmus et al. (2007); comparative information for VicPac2.0.2 is presented in the two far right columns.*

### Genome Annotation

Altogether, preliminary annotation predicted 42,389 genes in the alpaca genome. Of these, 22,462 were coding genes with an average of 14.7 exons per gene. Single-exon genes accounted for 11% (2,519) of these, thus multi-exon genes had an average of 16.5 exons. Overall, the predicted protein coding genes represented 39.6% of assembled sequence with coding exons covering 2.4% of assembled sequence. Coding genes contained 1.3 isoforms on average with a total of 29,337 coding isoforms. The number of genes predicted in the alpaca genome was higher than expected for a vertebrate or mammalian genome (Pennisi, 2012). This may be due to the limited transcriptome depth used to stitch exons into contiguous genes. The human transcriptome now has many terabases of sequencing depth from multiple tissues and conditions to generate a comprehensive (but still incomplete) transcriptome (Steward et al., 2017). The total number of predicted exons in the alpaca is also larger than more comprehensively annotated mammalian genomes, owing to small exonic fragments which may actually be exon extension and truncation events rather than unconnected exons. However, 22,462 predicted coding genes (*e*-value < 1e-20) are similar to the number of known proteins in the mammalian RefSeq database (Pruitt et al., 2014)^8^. It is possible that the remaining 19,927 predicted genes which have no similarity to any mammalian peptide, may be long non-coding RNA genes, having canonical intron-exon structure, polyadenylation sites and other coding gene-like features, but having a degenerate, or vestigial open reading frame sufficient to avoid nonsense mediated decay (Chang et al., 2007).

**FIGURE 1:**
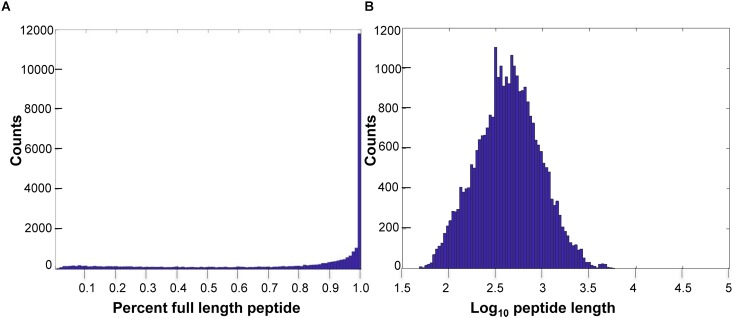
Predicted peptide length distributions. **(A)** Histogram of *VicPac3.1* peptide annotation showing number of peptides full length, as defined by 90% or better overlap of the closest human peptide match. **(B)** Histogram of predicted peptide lengths (log10) for *VicPac3.1*.

Of the predicted peptides, 58% were considered full length, compared to the length of the human best matching peptide, though more transcriptome sequencing is required to improve exon connectivity (Figure 1). Additionally, using OrthoFinder (Emms and Kelly, 2015), which identifies both “orthogroups” (genes descended from a single gene in the last common ancestor of a group of species, allowing many-to-many relationships and gene expansions) and orthologs between each pair of species in the comparison, we identified 21,136 orthogroups between *VicPac3.1*, dromedary, wild and captive Bactrian camels, cow and sheep with a mean size of 9 genes per orthogroup (Supplementary Table 4). Of these, 17,916 orthogroups contained an alpaca (*VicPac3.1*) protein, which provides further evidence for the quality of these gene annotations. Notably, 15,777 orthogroups contained all six species and 3,959 orthogroups contained all six species and were comprised of single-copy genes (Supplementary Table 4). The latter is close to the 4,104 mammalian BUSCOs^9^. The quality of the assembly and annotation was further validated by aligning all alpaca orthologs from chromosomally anchored scaffolds to the dromedary, Bactrian camel, cattle and human genomes (Supplementary Tables 5, 6) and compiling conserved synteny data between alpaca-human and alpaca-cattle with 11,765 and 8,494 orthologs, respectively.

Repeat content in *VicPac3.1* was 33.5% and largely the same as in *Vipacos_V1* (Table 1). RepeatMasker^10^ based annotation (Supplementary Table 7) identified over 4.6 million repeat elements, with the most abundant class being LINEs (19.41%), followed by LTR elements (5.81%) and SINEs (3.79%), of which the vast majority were MIRs (3.25%). DNA transposons accounted for 3.25% of sequence and these were largely comprised of the hAT-Charlie superfamily (1.75%).

**FIGURE 2:**
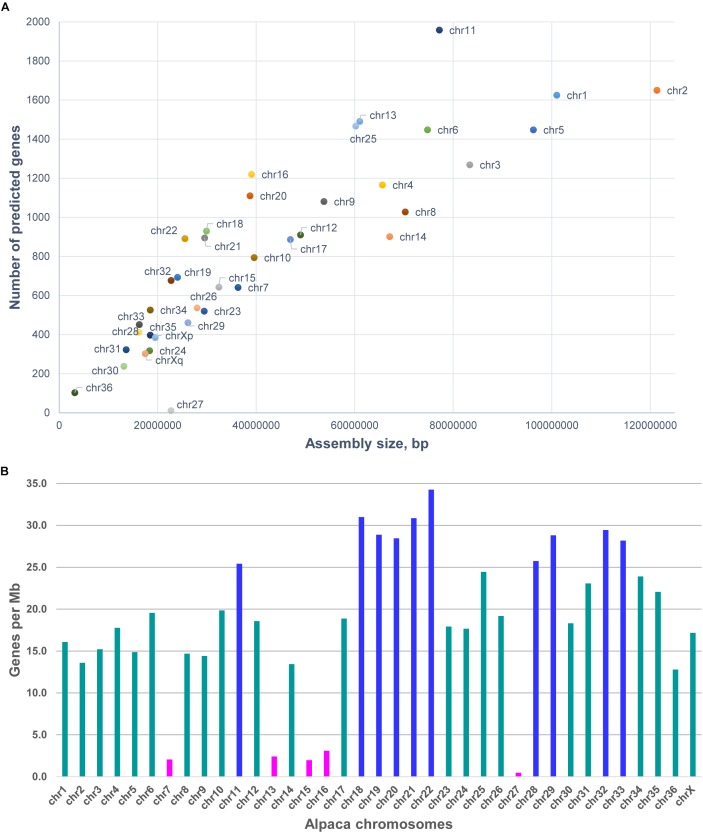
Features of alpaca chromosomes in *VicPac3.1*. **(A)** y-axis: the number of predicted genes per chromosome; x-axis: total length of chromosomally assigned scaffolds; **(B)** Gene density per chromosome; magenta – chromosomes with gene density < 5 genes/Mb; dark blue – chromosomes with gene density > 25 genes/Mb.

As 75.9% of scaffolds were chromosomally assigned, annotation this gave a better idea about the gene content and gene density of individual chromosomes (see Table 3 and Figure 2). In total, 31,748 predicted genes were assigned to chromosomes in *VicPac3.1*. The highest number was assigned to chr11 (1,958 genes), followed by chr2 and chr1 (Table 3 and Figure 2A). Chromosomes 1 and 2 had the longest assemblies, thus it was unsurprising that they contained the most predicted genes. On average, there was a predicted gene every 103 kb of chromosomally assigned sequence, with chr22 being the most gene dense (34.3 genes/Mb) while the most gene sparse chromosome was chr27 with 0.5 genes per Mb (Table 3 and Figure 2B). These numbers, however, are expected to change when the annotation improves and more of the currently unassigned scaffolds will be mapped to chromosomes.

### Highlights of Selected Features of the Alpaca Genome

#### The Major Histocompatibility Complex (MHC)

We specifically examined the sequence of the alpaca MHC and characterized MHC organization and gene content in relation to other camelids and cetartiodactyls. The region encodes many proteins of the innate and adaptive immune systems and contains the key immune response genes for host-pathogen interactions (Trowsdale, 1995; Kelley and Trowsdale, 2005; Plasil et al., 2016; Viluma et al., 2017). In order to counteract the high variability of pathogens and pathogen-derived molecules, the MHC has evolved into one of the most gene rich, highly polymorphic, and structurally complex regions of the mammalian genome, and is characterized by copy number variations and segmental duplications (Kelley and Trowsdale, 2005; Viluma et al., 2017). This complexity means that this relatively small region, which spans only approximately 4 Mb (Kelley and Trowsdale, 2005), is among the most challenging regions for genome assembly and annotation.

**FIGURE 3:**
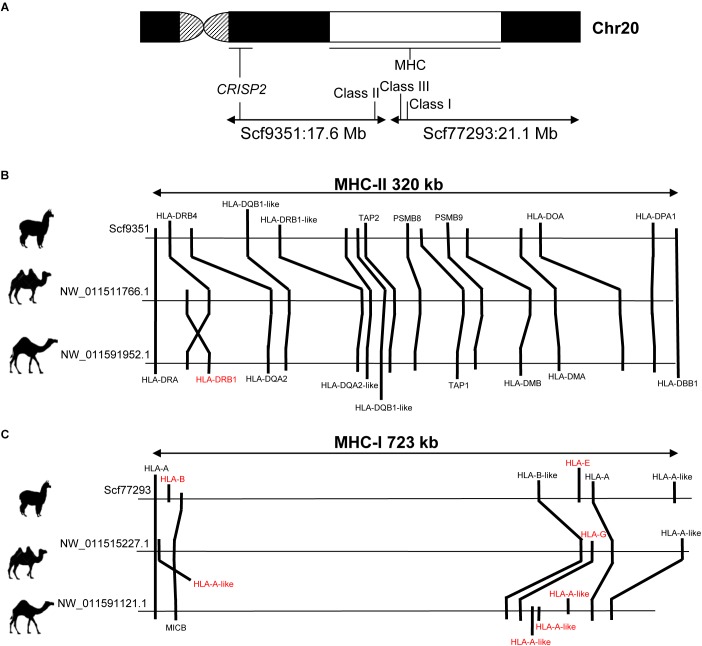
Comparison of MHC in alpaca, dromedary and Bactrian camel. **(A)** Schematic of camelid chr20 showing FISH map positions of MHC and *CRISP2* and corresponding scaffolds in *VicPac3.1*; **(B)** comparative organization of MHC Class II; **(C)** comparative organization of MHC Class I; gene symbols in red denote loci that show differences between species; relative positions of genes and distances between loci correspond to the sequence map.

The MHC is located in camelid chr20, as revealed by cytogenetic mapping of specific MHC loci in alpaca (Avila et al., 2014a) and dromedary (Plasil et al., 2016). In *VicPac3.1*, two large scaffolds of 21.1 Mb (ScfyRBE_77293) and 17.6 Mb (ScfyRBE_9351) (Table 3 and Supplementary Table 2) were uniquely mapped to chr20. This resulted in a 38.7 Mb assembly for chr20, making it among the largest and most contiguous assemblies of the medium size alpaca chromosomes (Table 3). The assembly of chr20 in *VicPac3.1*, also served as a good scaffold for placing dromedary and Bactrian camel assemblies on the chromosome, allowing for detailed comparison of the MHC region in New and Old World camelids (Figure 3).

The alpaca MHC spanned two separate scaffolds: Class I (723 kb) and Class III were in ScfyRBE_77293 and Class II (320 kb) was in ScfyRBE_9351 (Figure 3). The overall organization of the alpaca MHC relative to the centromere-telomere axis closely resembled the MHC of the dromedary and Bactrian camel (Plasil et al., 2016), i.e., centromere-Class II-Class III-Class I-telomere (Figure 3A). This orientation was confirmed with the cytogenetic and sequence map position of the *CRISP2* gene, which does not belong to MHC, maps very close to the centromere in chr20q (Avila et al., 2014a), and was found in scaffold ScyRBE_9351 together with MHC Class II sequences (Figure 3A). The MHC organization, like that seen in camelids where all MHC genes are syntenic and Class III sequences are positioned between Class I and Class II sequences, is typical of all mammalian (Ruan et al., 2016a,b; Viluma et al., 2017) and many vertebrate species (Flajnik, 2018). Alpaca and camel Class II sequences seem to be present in one block as seen in humans, pigs and horses (Li et al., 2012; Viluma et al., 2017). This is in contrast to cattle, sheep and porpoise where Class II has been disrupted by a large inversion into IIa and IIb sub-regions that happened in the ancestral chromosome of these cetartiodactyl lineages (Childers et al., 2006; Gao et al., 2010; Li et al., 2012; Ruan et al., 2016a).

Further, we more closely inspected the gene contents and order of Class I and Class II genes in alpaca, Bactrian camel and dromedary (Figure 3). In general, these MHC regions were collinear in camelids, though a few differences between the species were observed. In Class I, seven genes were annotated in alpaca and Bactrian camel but nine genes in the dromedary, because of an expansion of *HLA-A*-like sequences in the latter (Figure 3C). We speculate that the unique and specialized microbiomes of deserts (Bang et al., 2018) may have driven expansion of HLA-A in this genome. Further, no sequences of *HLA-G* corresponding to Class I heavy chain paralogs were found in alpaca, though these sequences are present in both camel species. In contrast Class I heavy chain paralogs *HLA-E* and *HLA-B* were present only in alpaca and not in camels. Class II contained 16 genes in alpaca and 17 genes in camels (Figure 3B). The difference was due to the *HLA-DRB1* locus, which was found in camels but not in the alpaca. Furthermore, an inversion has probably happened in the dromedary Class II changing the relative order of *HLA-DRB1* and *HLA-DRB4* in relation to that in the Bactrian camel. These minor differences in MHC gene content between camelids may be true, though it is equally plausible that they are due to difficulties associated with annotation of the highly variable MHC sequences. However, a general observation about the camelid Class II region was that even though the region is collinear between the three species, the relative positions of genes and distances between them are similar in camels but different in alpacas (Figure 3B), suggesting possible independent segmental duplications or expansion of gene families in the two camelid lineages.

#### Chromosome 36 – A Candidate Region for the Minute Chromosome Syndrome (MCS)

Chromosome 36 is the smallest autosome in the alpaca genome and of interest for several reasons. First, it was the only chromosome in camelid genomes for which human and other mammalian homology could not be revealed by Zoo-FISH (Balmus et al., 2007). Because of that, it was suggested that the chromosome is largely heterochromatic and contains very few genes if any at all. This hypothesis changed when two protein coding genes, *ZPBP* and *VWC2*, were mapped to chr36 by FISH revealing its homology to a segment in the short arm of human chr7 (HSA7p) at 49–56 Mb (Avila et al., 2014a, 2015). The second and perhaps even more important reason for interest in chr36 is its involvement in the *Minute Chromosome Syndrome (MCS)* (Avila et al., 2015). This is a unique chromosomal abnormality in alpacas and llamas with no counterpart in any other mammalian species. The condition is invariably associated with abnormal sexual development and infertility in females and curiously, it is recurrent (Avila et al., 2014b; Fellows et al., 2014; Raudsepp and Chowdhary, 2016). Cytogenetic manifestation of *MCS* is a dramatic size difference between the homologs of chr36, and because it was thought that the smaller homolog is abnormal, the condition was named *minute.* Now it is known that the abnormal one is the larger chr36, because it carries a massive nucleolus organizer region (NOR), which is not found in chr36 in normal alpacas (Avila et al., 2015). Molecular causes of *MCS* are, however, poorly understood and an improved reference assembly for chr36 is an important resource to study the condition at molecular level. In *VicPac3.1*, chr36 is represented by three scaffolds with a total size of about 5 Mb (Table 4). Altogether, we predicted 64 genes in chr36, of which 23 have known orthologs in HSA7p showing that alpaca chr36 shares homology to a ∼12 Mb region in HSA7p (Table 4).

**Table 4:** Chromosome 36 scaffolds and predicted genes with orthologs in HSA7.

Scaffold	Scaffold size, bp	Gene symbol	HSA7 sequence position
ScfyRBE_2631	352,287	*IGFBP1*	chr7:45,888,357-45,893,668
		*IGFBP3*	chr7:45,912,598-45,921,274
ScfyRBE_77331	2,828,351	*TNS3*	chr7:47,275,154-47,582,144
		*HUS1*	chr7:47,963,288-47,979,543
		*SUN3*	chr7:47,987,151-48,029,119
		*C7orf57*	chr7:48,035,520-48,061,297
		*ABCA13*	chr7:48,171,460-48,647,495
		*VWC2*	chr7:49,773,661-49,921,950
		*ZPBP*	chr7:49,937,441-50,093,264
		*SPATA48*	chr7:50,096,036-50,159,830
		*IKZF1*	chr7:50,304,669-50,405,101
		*FIGNL1*	chr7:50,444,129-50,542,535
		*DDC*	chr7:50,531,759-50,543,463
		*GRB10*	chr7:50,592,580-50,782,567
		*COBL*	chr7:51,016,212-51,316,799
ScfyRBE_77323	2,197,127	*VSTM2A*	chr7:54,542,325-54,571,080
		*SEC61G*	chr7:54,752,253-54,759,974
		*LANCL2*	chr7:55,365,448-55,433,742
		*VOPP1*	chr7:55,470,613-55,572,525
		*PGAM2*	chr7:44,062,727-44,065,587
		*DBNL*	chr7:44,044,717-44,061,716
		*URGCP*	chr7:43,875,913-43,906,626
		*MRPS24*	chr7:43,866,558-43,869,557


*The order of genes in the table follows their relative order within chr36 scaffolds*.

#### Adaptations to High Altitude

Alpacas are adapted to high altitude and low oxygen environments, and therefore different evolutionary forces must have shaped their genomes as compared to dromedary and Bactrian camels, the desert species. Therefore, we specifically aimed to identify in the alpaca genome candidate genes for high altitude adaptations. We selected 20 genes for which signatures of positive selection have been reported in other high altitude species (Table 5). Through the application of *d*_N_/*d*_S_ substitution ratio *ω* (see Material and Methods; Supplementary Table 8), we investigated whether any of these genes exhibit signals of selection in camelids. Nine high altitude adaptation genes exhibited sites that were under negative (purifying) selection in the alpaca compared to other camelids (Table 5), suggesting selection to remove deleterious mutations that might alter gene function. Three genes in this group, *EPAS1, EGLN1*, and *PPARA* regulate or are regulated by hypoxia inducible factor 1α (Hif-1α), which is a master regulator of the cellular response to hypoxia (Qiu et al., 2012; Simonson et al., 2012). All three genes are known to be involved in high altitude adaptations in dogs (Gou et al., 2014), humans (Beall, 2014; Bigham and Lee, 2014; Jeong et al., 2014), and *EPAS1* also in Tibetan snakes (Li et al., 2018). *EPAS1* genotypes have been associated in several studies with the dampened hemoglobin phenotype, while noncoding variants in and around *EPAS1* and *EGLN1*, are strongly associated with a reduced blood concentration of hemoglobin in Tibetan highlanders (Beall, 2014; Bigham and Lee, 2014; Gou et al., 2014). Under purifying selection in alpacas are also genes encoding for ARG2 and ADAM17 proteins, which both affect Hif-1α stability and activity (Qiu et al., 2012). Alleles of human *ADAM17* are present at significantly different frequencies in Tibetans compared to low-altitude dwellers (Simonson et al., 2012). The *NFE2L2* gene has unique amino acid residue changes in the dromedary and Bactrian camel genomes and is correlated with the oxidative stress response (Wu et al., 2014). In the present analysis, this gene exhibits signatures of purifying selection in alpaca, but not in camels (Table 5 and Supplementary Table 8). Among the candidate genes tested, only *PPARA* and *EHHADH* were under positive selection in alpacas but not in camels, showing significant higher branch specific *ω* value (Supplementary Table 8). Signatures of both purifying and positive selection were found in different regions of *NOTCH4 and RBPJ*, with both genes suggested to be involved in the regulation of responses to hypoxia in deer mouse (Scott et al., 2015) and pig (Jia et al., 2016).

**Table 5:** Candidate genes for high altitude adaptations and signatures of selection in the alpaca.

Gene symbol	Signature of selection	Species where the gene is under positive selection	References
ACAA1A	–	Deer mouse	Scott et al., 2015
ADAM17	Negative	Yak	Qiu et al., 2012
ARG2	Negative	Yak	Qiu et al., 2012
ATF6	–	Pig	Jia et al., 2016
CKMT1	–	Deer mouse	Scott et al., 2015
EFEMP1	Negative	Pig	Jia et al., 2016
EGLN1	Negative	Yak, dog, human	Qiu et al., 2012; Bigham and Lee, 2014; Gou et al., 2014; Jeong et al., 2014
EHHADH	Positive	Deer mouse	Scott et al., 2015
EPAS1	Negative	Dog, human, snakes	Bigham and Lee, 2014; Gou et al., 2014; Jeong et al., 2014; Li et al., 2018
ERP44	–	Camels (oxidative stress	Wu et al., 2014
HOXB6	–	Pig	Jia et al., 2016
IKBKG	–	Pig	Jia et al., 2016
KLF6	Negative	Pig	Jia et al., 2016
MGST2	–	Camels (oxidative stress	Wu et al., 2014
MMP3	–	Yak	Qiu et al., 2012
NFE2L2	Negative	Camels (oxidative stress	Wu et al., 2014
NOTCH4	Negative and positive	Deer mouse	Scott et al., 2015
PPARA	Positive	Deer mouse, human	Simonson et al., 2012; Scott et al., 2015
RBPJ	Negative and positive	Pig	Jia et al., 2016
SF3B1	Negative	Pig	Jia et al., 2016


#### Genes Involved in Fiber Color and Quality

Alpacas produce one of the most highly prized natural fibers in the world. This fiber comes in a large range of natural colors, which is a significant point of differentiation with fine fiber sheep, such as Merinos. The key mammalian color genes *MC1R* (melanocortin 1 receptor) and *ASIP* (agouti signaling protein) have also been found to regulate alpaca fiber color (Feeley and Munyard, 2009; Feeley et al., 2011). Interestingly, although the donor of the DNA used for the alpaca genome (*Carlotta*; Table 1) was fawn, the *ASIP* gene in all *VicPac* genomes, including *VicPac3.1*, contains a 57 bp deletion in exon 4 associated with loss of function of ASIP, and black color. However, this is counter-acted by epistatic interaction of *MC1R*, which is homozygous for the alternative allele at two of the three known loss of function SNPs (Feeley and Munyard, 2009), and which prevents the expression of eumelanin (black pigment). Importantly, *MC1R* is correctly annotated in *VicPac3.1* vs. *VicPac2*, in which it was misnamed and annotated as having three exons instead of one. It was recently shown that alpaca and camel *MC1R* maps by FISH to chr21 (Alshanbari et al., 2019) and not to chr9 as anticipated by Zoo-FISH (Balmus et al., 2007) and FISH mapping orthologs from HSA16q (Avila et al., 2014a). The location of *MC1R* in alpaca chr21 is supported by *VicPac3.1*, showing that chr21 shares conserved synteny with both HSA1 and the terminal part of HSA16q (Table 6). The annotation of other important mammalian color genes such as Tyrosinase related protein 1 (*TYRP1*), dopachrome tautomerase (*DCT*), Premelanosomal protein (*PMEL*), KIT oncogene (*KIT*), KIT oncogene ligand (*KITLG*), and Solute carrier 36 A1 (*SLC36A1*), is also improved in *VicPac3.1* as compared to *VicPac2* (Table 7).

**Table 6:** Alpaca chr21 scaffolds and predicted genes (*MC1R* is highlighted) with known human orthologs.

Scaffold	Gene symbol	Human location; chr: sequence position
ScfyRBE_283	*NOS1AP*	chr1:162,069,774-162,368,451
	*DDR2*	chr1:162,632,465-162,787,400
	*TOR3A*	chr1:179,081,377-179,095,996
ScfyRBE_77299	*XPR1*	chr1:180,632,004-180,890,251
	*LAMC2*	chr1:183,186,288-183,244,900
	*EDEM3*	chr1:184,690,231-184,754,913
ScfyRBE_77374	*PIEZO1*	chr16:88,715,338-88,785,220
	*ZFPM1*	chr16:88,453,317-88,537,016
	*BANP*	chr16:87,951,434-88,077,318
	*IRF8*	chr16:85,898,803-85,922,609
	*GSE1*	chr16:85,613,216-85,676,204
	*FMO5*	chr1:147,186,259-147,225,638
	*CTSK*	chr1:150,796,208-150,808,323
	*NIT1*	chr1:161,118,101-161,121,067
ScfyRBE_14	*GAS8*	chr16:90,022,600-90,044,971
	*MC1R*	chr16:89,914,847-89,920,951
	*SPG7*	chr16:89,490,917-89,557,748
	*ANKRD11*	chr16:89,285,175-89,490,318
	*SLC22A31*	chr16:89,195,761-89,201,664


*The order of genes in the table follows their relative order in corresponding scaffolds.*

**Table 7:** Select mammalian coat color genes in *VicPac3.1.*

Gene symbol	*VicPac3.1* no. of exons	*VicPac2.0* no. of exons	Scaffold *VicPac3.1*	Alpaca chr. *VicPac3.1*	FISH; Avila et al., 2014a
*MC1R*	1	3	ScfyRBE_14	21	n/a
*TYRP1*	8	7	ScfyRBE_2524	4	4q21-q22
*DCT*	8	7	ScfyRBE_4179	14	n/a
*PMEL*	12	7	ScfyRBE_77320	16	n/a
*KIT*	22	19	ScfyRBE_26	2	2q24
*KITLG*	10	11	ScfyRBE_77306	12	12q22
*SLC36A1*	14	10	ScfyRBE_5827	3	3q12


The new assembly also improved sequence quality, annotation and chromosomal assignment of keratin (*KRT*) and keratin-associated protein (*KRTAP*) genes, some of which are the primary candidates for fleece and fiber quality (Allain and Renieri, 2010). Like in other mammals, alpaca *KRT* and *KRTAP* genes are clustered in gene families and located predominantly in chr12 (25 *KRT* genes) and chr16 (22 *KRT* genes and 2 *KRTAP*s) (Table 8).

**Table 8:** Clusters of keratin and keratin-associated protein genes in *VicPac3.1.*

Gene symbol	*VicPac3.1* Scaffold	Chromosome
*KRT18; KRT8; KRT78; KRT79; KRT4; KRT3; KRT77; KRT1; KRT2; KRT73; KRT72; KRT74; KRT71; KRT5; KRT6A; KRT75; KRT82; KRT84; KRT85; KRT83; KRT86; KRT81; KRT82; KRT7; KRT80*	ScfyRBE_77306	12
*KRT17; KRT16; KRT14; KRT9; KRT19; KRT13/15; KRT36; KRT35; KRT32; KRT31; KRT33A; KRTAP3-1; KRTAP3-3; KRT40; KRT39; KRT23; KRT20; KRT12; KRT27; KRT26; KRT25; KRT24; KRT222;*	ScfyRBE_77388	16
*KRT6C; KRT6B*	ScfyRBE_2857	n/a
*KRTAP13-1; KRTAP13-2; KRTAP7-1*	ScfyRBE_4	1


*Genes are ordered in the table following their relative order in corresponding scaffolds.*

### Summary

Reference assembly *VicPac3.1* with its improved accuracy, contiguity, chromosomal anchoring and preliminary annotation, constitutes a key resource for understanding the architecture of the alpaca genome, and is critical for the discovery of genetic blueprints of diseases/disease resistance, congenital disorders and traits of biological significance. It will provide a strong basis for whole genome population-scale studies in alpacas and other South American camelids, and for comparative genomics among camelids and with other mammals. High quality assembly is also the prerequisite for in depth functional annotation of the alpaca genome in the future, similar to the FAANG initiatives that are ongoing in other domestic species (Andersson et al., 2015).

## Materials and Methods

### Ethics Statement

Procurement of blood and tissue samples followed the United States Government Principles for the Utilization and Care of Vertebrate Animals Used in Testing, Research and Training. These protocols were approved as AUP #2011-96, # 2018-0342 CA and CRRC #09-47 at Texas A&M University.

### Samples and DNA Isolation

Peripheral blood was procured from a female Huacaya alpaca *Nyala’s Accoyo Empress Carlotta* – the same DNA donor that was used for the assemblies VicPac1 and VicPac2. Blood DNA was isolated using a Gentra Puregene Blood Kit (Qiagen), following the manufacturer’s protocol, and evaluated for quality and quantity on the Agilent 2200 TapeStation. This showed that the gDNA was of high quality and high molecular weight (i.e., fragments larger than 50,000 bp) and suitable not only for Illumina sequencing, but also for both long-read Pacific Biosciences (PacBio) sequencing and constructing Chicago^®^ libraries for HiRise^TM^ scaffolding (Dovetail Genomics).

### Genome Sequencing and Assembly

Two sequencing libraries were generated: a shotgun paired –end library (fragment size 200 bp) and a mate-pair library (2–5 kb) which were sequenced across 8 lanes on the Illumina 2500 platform (5 lanes paired-end and 3 lanes mate-pair; both 2 × 100 bp) commercially at Macrogen Inc. (SK) to generate short-read sequence data with > 100X genome coverage. Additionally, one PacBio SMRT cell library was constructed and sequenced across 20 SMRT cells on the RSII platform to generate long-read data with 5-6X genome coverage; conducted commercially by PacBio Sequencing Services at the University of Washington. Short reads were filtered for quality, adaptors removed and filtered for a minimum length of 60 bp using Trimmomatic v0.33 (Bolger et al., 2014) with ILLUMINACLIP:NexteraPE-PE.fa:2:30:10 SLIDINGWINDOW:4:25 MINLEN:60. The final genome assembly was produced through a multi-stage process. First, we generated a hybrid assembly with MaSuRCA v3.2.1 (Zimin et al., 2013) using default parameters, using all the paired-end and mate-pair Illumina data, ∼22X Roche 454 data from *VicPac2.0* and ∼3X Sanger data from *VicPac1* (both available via PRJNA30567). The hybrid assembly was designated as *Qmas1*. This was further developed into a meta-assembly guided by the *VicPac2* assembly and Illumina mate-pair alignments using Metassembler (Wences and Schatz, 2015) with MUMmer4 (Marcais et al., 2018) and the following parameters: *MateAn_A = 2000, MateAn_B = 3000, nucmer_l = 50, nucmer_c = 300*) in order to generate a more contiguous assembly for the alpaca genome. This assembly was designated as *Qmas1/VicPac2*. Using the ∼5X PacBio consensus sub-reads and Illumina mate-pair data, we scaffolded the *Qmas1/VicPac2* contigs using OPERA-LG v.2.05 (Gao et al., 2016); assembly designation *VicPac3.0*. The final assembly, *VicPac3.1*, was obtained by constructing two 2X 151 bp Chicago^®^ libraries which were then subjected to HiRise^TM^ scaffolding along with *VicPac3.0* and the mate-pair libraries, and this was done by Dovetail Genomics (United States).

#### Chromosomal Assignment

Sequence scaffolds were anchored to alpaca autosomes and the X chromosome with the help of alpaca cytogenetic markers (Avila et al., 2014a,b, 2015). Sequences of the overgo and PCR primers that were used for FISH analysis were mapped to *VicPac3.1* scaffolds using BBMap v35^11^ with the following parameters: *pairedonly = t, minid = 0.97 and pairlen = 500*, which generated no ambiguous mappings, retaining those with 97% identity (equivalent of 1 bp mismatch) and that the primers were mapped in the correct orientation. Overgo primers were mapped with the same parameters, but allowing for 95% identity matches. We considered scaffolds anchored when the primers uniquely mapped to one scaffold and the primers mapped in the correct orientation.

#### Preliminary Genome Annotation

A preliminary annotation of the *VicPac3.1* was produced primarily for comparative assessment. We used AUGUSTUS v3.3.1 (Hoff and Stanke, 2018) for gene model prediction. First, gene hints were built using VicPac2.0.2 (GCA_000164845.3), human (GCA_000001405.27), cow (*Bos taurus*; GCA_000003055.3), dromedary (*Camelus dromedaries*; GCA_000767585.1), and Bactrian camel (GCF_000311805.1 and GCF_000767855.1) peptides. Peptides were mapped to the alpaca genome assembly using BLAT v. 36x2 (Kent, 2002) with default parameters, then converted to hints with blat2hints.pl, taking the best two matches to every peptide. AUGUSTUS was then run with these hints, and human was used as the training species (closest pre-trained species). Finally, the predicted gene models were confirmed by mapping testis and skin RNA-Seq data to *VicPac3.1* with STAR v 2.5 (Dobin et al., 2013) in two-pass mode with default parameters and checking correct junctions in the STAR junction files and well-known gene models in IGV (Robinson et al., 2011; Thorvaldsdottir et al., 2013).

Interspersed repeats were identified through a homology-based approach using RepeatMasker v4.07^12^ with RMBlast v 2.2.27+^13^ and TRF v4.09 (Benson, 1999) searches against Dfam 2.0, Dfam consensus (Hubley et al., 2016) and Repbase (Bao et al., 2015) databases, both 20170127 issue.

#### Genome Contiguity and Completeness Assessment

The contiguity and completeness of the alpaca genome assemblies were evaluated using several methods. We computed core assembly metrics (N50, L50, number of scaffold, longest scaffold, GC content and proportion N’s) for our 4 assemblies, *VicPac2* and the alpaca assembly (Vi_pacos_V1) (Wu et al., 2014). Completeness of the four assemblies generated in this study was directly compared using BUSCO (Simao et al., 2015) analysis of conserved orthologs. BUSCO score comparisons between organisms can serve as useful benchmarks for assembly completeness so we also compiled BUSCO assessments for cow (v3.1.1; GCF_000003055.6), sheep (*Ovis aries*; v4.0; GCF_000298735.2) dromedary (GCF000767585.1), and Bactrian (both domesticated, GCF_00767855.1, and wild, GCF_000311805.1) camels. We ran BUSCO v3.0.2^14^ with *geno* mode, mammalia_odb9, Blast v2.2.26+ (Camacho et al., 2009), HMMer v3.1 (Eddy, 2011) and Augustus v3.2. Lastly, we compared gene model predictions at the protein level for *VicPac3.1, VicPac2*, and Vi_pacos_V1 using both standard and reciprocal best-hit blastp, using Blast v2.2.26+ and an evaluated cut off of e^-10^.

#### Comparative Analysis

We used OrthoFinder v1.1.10 (Emms and Kelly, 2015) with Diamond v0.9.9.110 (Buchfink et al., 2015), and FastME v2.1.5 (Lefort et al., 2015) to identify orthologs, orthogroups, paralogs and compute gene (ortholog) and species trees in an all vs. all comparison of *VicPac3.1*, dromedary, and both wild and domesticated Bactrian camels, cow and sheep.

The *VicPac3.1* scaffolds anchored to chr20 were used to anchor dromedary and Bactrian scaffolds to chr20, using reciprocal best-hit Blast v2.2.26+, blastn implemented with default settings. Pairwise comparative alignments were conducted for anchored chr20 scaffolds using MAUVE v 2.4.0 (Darling et al., 2004) with default settings for alpaca (*VicPac3.1*) vs. dromedary, alpaca vs. Bactrian, alpaca vs. cow and alpaca vs. sheep. Cow and sheep genome assemblies are already chromosomally assigned so we used their respective chr20 sequence fasta. We compared Major Histocompatibility complex (MHC) gene synteny among camelid chr20 (alpaca, dromedary and Bactrian camels), using orthology and syntenic position between anchoring orthologs. All MHC and MHC-like genes (any gene with a blast *e*-value less than 1e-20 to any human MHC gene) in the MHC Class I and MHC Class II syntenic regions were annotated with respect to human peptide best matches.

#### Positive Selection

To investigate whether candidate genes involved in adaptation to high altitudes exhibit signals of selection among the Camelidae, coding sequences (CDS) of 23 genes previously identified as potentially having a role in high altitude adaptation (*EGLN1, EPAS1, PPARA, IKBKG, KLF6, RBPJ, SF3B1, EFEMP1, HOXB6, ATF6, ADAM17, MMP3, ARG2, ERP44, NFE2L2, MGST2, AQP1, AQP2, AQP3, CKMT1, EHHADH, ACAALA, NOTCH4)* were extracted from the *VicPac3.1* and from dromedary and wild and domesticated Bactrian camel assemblies. Multiple sequence alignments were conducted using GUIDANCE2 (Sela et al., 2015) with default quality cutoffs, codon alignments with PRANK (Loytynoja and Goldman, 2010) as the MSA program specified with the –F parameter. We used the longest CDS of a gene for alignment when there was more than one per species. Signatures of selection were searched with two *d*_N_/*d*_S_ based tests using HyPhy^15^3pc (Pond et al., 2005). First, the aBSREL (Smith et al., 2015) branch-site model, which tests if each branch in the phylogeny has a proportion of sites evolving under positive selection, as we tested all branches we FDR corrected the likelihood-ratio test *p*-values. Second, the FEL (Kosakovsky Pond and Frost, 2005) model which assumes selective pressure is constant for each site across the phylogeny and calculates whether the nonsynonymous (*d*_N_) substitution rate is significantly different from the synonymous (*d*_S_) rate, using the likelihood ratio test.

#### Transcriptome Sequencing and Analysis

High quality (RIN > 9.6) RNA was extracted from the testis of one normal male alpaca and one normal male llama using PureLink RNA Mini Kit (Ambion). The RNA was converted into cDNA with NEXTflex Rapid Directional qRNA-Seq kit (BIOO), prepared into 2 × 100 bp PE TruSeq libraries (Illumina), and sequenced on Illumina HiSeq2500 platform. We obtained, on average, 90 million PE reads per sample. The RNA from the skin samples was prepared as reported in Cransberg Ph.D. Thesis (Cransberg, 2017). Briefly, skin biopsies were collected from 20 white, 20 brown and 5 black alpacas, the RNA was extracted using Trizol reagent and the FastPrep system (Thermo Life Sciences) and an RNeasy Kit (Qiagen). After confirmation of RNA quality (Bioanalyser; Agilent) three equi-molar pools of RNA were prepared (one for each color). Sequencing libraries were prepared using an Illumina Tru-seq RNA kit, and sequenced on a single lane of an Illumina Genome Analyser GAIIx to generate 54 bp PE reads.

#### Genome Size Estimation

Genome size was first estimated using filtered short-read *k*-mer distributions. *k*-mer frequencies were calculated using Jellyfish v2.2.8 (Marcais and Kingsford, 2011) with canonical *k*-mers, for a range of *k*-values (17, 21, 25, and 31). These *k*-mer distributions were then analyzed in Genoscope^16^ (Vurture et al., 2017) with a maximum *k*-mer coverage of 1,000 and –1 (where –1 is no maximum coverage). Genome size was also estimated by flow cytometry using the protocol described elsewhere (Zhu et al., 2012) with a modification that the concentration of RNAse was doubled (200 μg/ml). Briefly, primary fibroblast cell lines of 2 alpacas and 2 horses were cultured in T25 culture flasks until 100% confluency. The cells of each individual were trypsinized, washed 6 times in PBS, fixed in cold 70% ethanol and stained with Propidium Iodine. The stained cells were analyzed on a BD Accuri^TM^ C6 personal flow cytometer separately for each animal. Results were gated in order to prevent exogenous DNA from lysed cells from affecting the results. Peaks were observed based on the amount of PI absorbed by each cell population (Supplementary Figure 2). Both horses and one alpaca were measured on 3 separate occasions, the other alpaca was only measured once due to a limited number of cells. The average median PI concentration and a 95% CI was calculated for the horse an alpaca, respectively, using all measurements available. The genome size was estimated using the formula: Size_alpaca_ = PI_alpaca_/PI_horse_ (2.7 Gb). Where PI_alpaca_ denotes the median amount of PI absorbed by alpaca cells; PI_horse_ denotes the median amount of PI absorbed by horse cells; 2.7 Gb is the expected size of the horse genome (Wade et al., 2009).

## Ethics Statement

Procurement of blood and tissue samples followed the United States Government Principles for the Utilization and Care of Vertebrate Animals Used in Testing, Research and Training. These protocols were approved as AUP #2011-96, # 2018-0342 CA and CRRC #09-47 at Texas A&M University.

## Author Contributions

TR, BA, and PP designed and initiated the project. MR, KM, LC, FJ, TA, FA, MJ, GW, RC, and TR conducted the experimental work. MR, KM, FJ, LC, and TA carried out the genome assembly, annotation, and bioinformatics analyses. MJ and FA contributed to the testis transcriptome and data analyses. TR, AT, RC, and KM collected the samples for genome and transcriptome sequencing. TR, MR, KM, and LC wrote the manuscript with input from all authors.

## Conflict of Interest Statement

The authors declare that the research was conducted in the absence of any commercial or financial relationships that could be construed as a potential conflict of interest. The handling Editor is currently organizing a Research Topic with one of the authors KM, and confirms the absence of any other collaboration.

## Footnotes

1 https://www.ncbi.nlm.nih.gov/

2 https://genome.ucsc.edu/

3 http://www.ensembl.org/index.html

4 https://busco.ezlab.org/

5 http://www.ensembl.org/index.html?redirect=no

6 https://genome.ucsc.edu/

7 https://www.ncbi.nlm.nih.gov/genome

8 https://www.ncbi.nlm.nih.gov/refseq/

9 https://busco.ezlab.org/

10 http://www.repeatmasker.org/

11 http://bib.irb.hr/datoteka/773708.Josip_Maric_diplomski.pdf

12 http://www.repeatmasker.org/

13 http://www.repeatmasker.org/RMBlast.html

14 https://vcru.wisc.edu/simonlab/bioinformatics/programs/busco/BUSCO_v3_userguide.pdf

15 http://www.hyphy.org

16 http://qb.cshl.edu/genomescope/
