# Supplementary material for: Chromosome-Level Alpaca Reference Genome VicPac3.1 Improves Genomic Insight Into the Biology of New World Camelids
Source: Front Genet. 2019 Jun 21;10:586. doi: 10.3389/fgene.2019.00586 (PMC6598621; doi:10.3389/fgene.2019.00586)
Supplement: Supplementary file 1 [file Image_1.pdf]

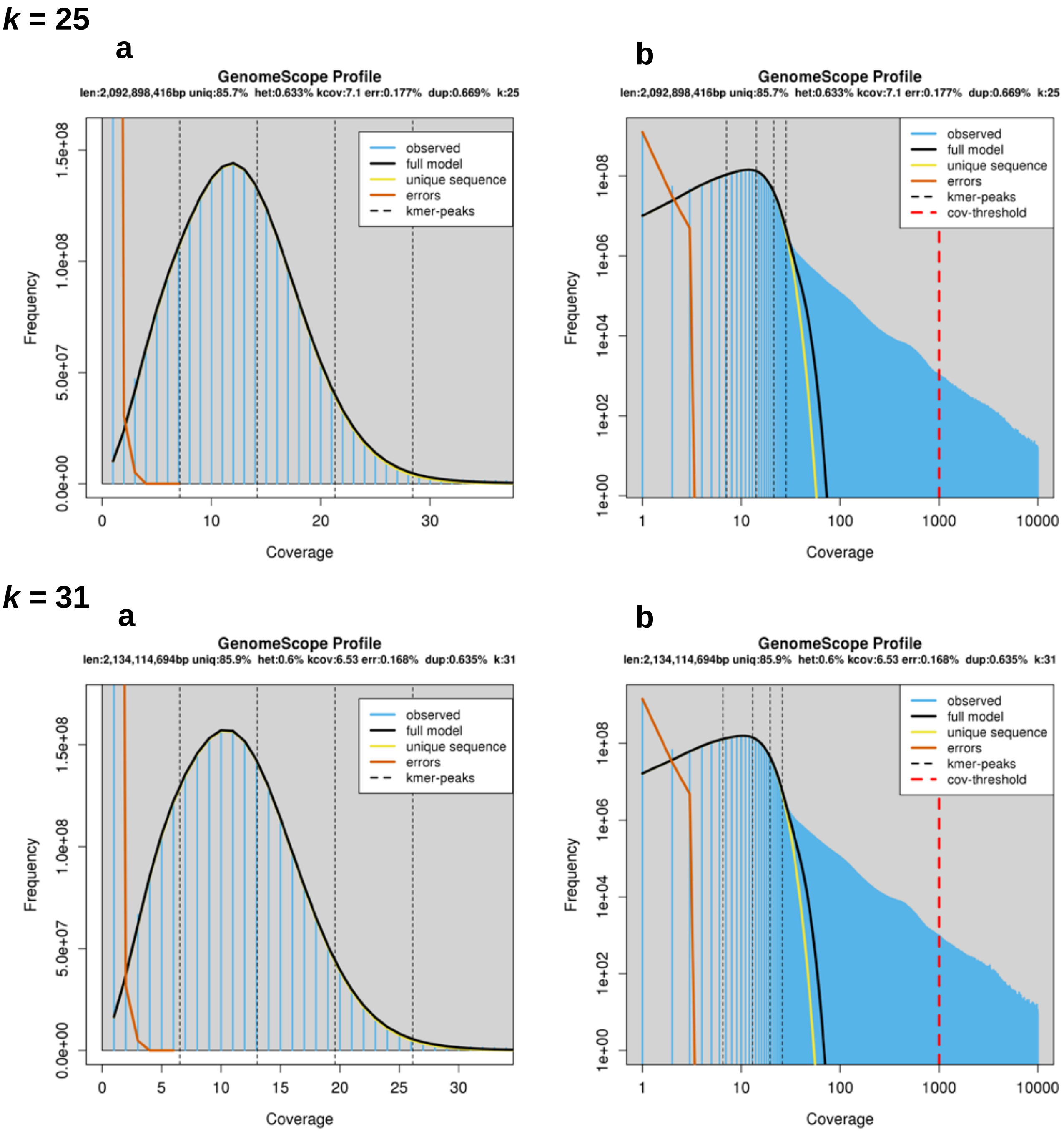

Supplementary Figure 1. Alpaca genome size by k-mer distributions. GenomeScope generated *k*-mer frequency (**a**) and log-transformed *k*-mer coverage profiles (**b**). Models produced for *k* = 21 and *k* = 31.
