# Supplementary material for: Chromosome-Level Alpaca Reference Genome VicPac3.1 Improves Genomic Insight Into the Biology of New World Camelids
Source: Front Genet. 2019 Jun 21;10:586. doi: 10.3389/fgene.2019.00586 (PMC6598621; doi:10.3389/fgene.2019.00586)
Supplement: Supplementary file 2 [file Image_2.pdf]

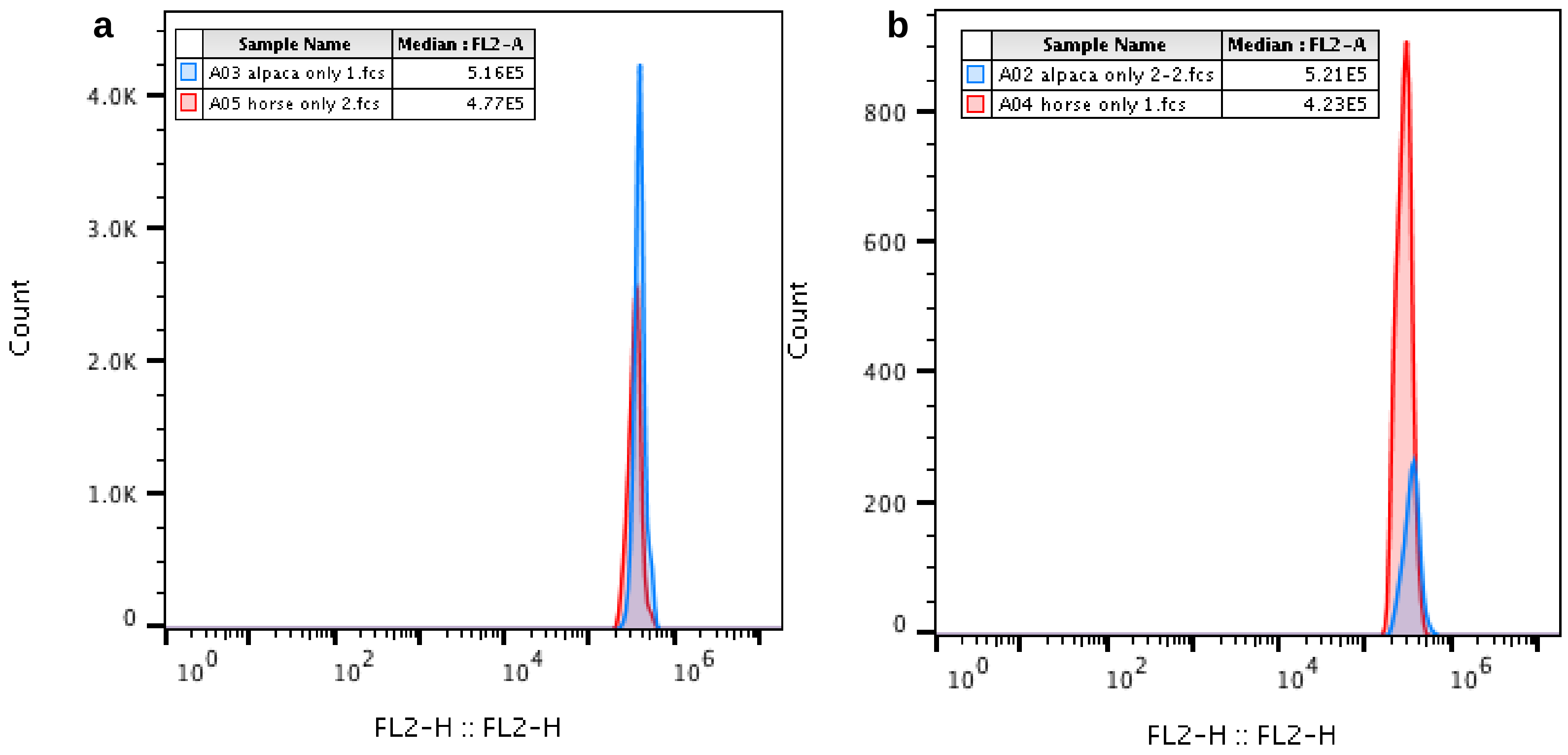

Supplementary Figure. 2. Alpaca genome size by flow cytometry. The y-axis shows the number of cells analyzed and the x-axis shows the concentration of propidium iodine (PI) in those cells. PI concentration was measured independently for each animal and then the graphs were overlaid. The average median PI concentration was calculated for both horse and alpaca. Genome size was estimated as a function of the ratio of the average median PI concentrations. (**a**) shows overlaid PI concentration peaks for alpaca 1 and horse 2. (**b**) shows overlayed PI concentration peaks for alpaca 2 and horse 1.
